# Microbial Community Establishment, Succession, and Temporal Dynamics in an Industrial Semi-Synthetic Metalworking Fluid Operation: A 50-Week Real-Time Tracking

**DOI:** 10.3390/microorganisms12020267

**Published:** 2024-01-26

**Authors:** Renuka Kapoor, Suresh Babu Selvaraju, Venkataramanan Subramanian, Jagjit S. Yadav

**Affiliations:** Department of Environmental and Public Health Sciences, Division of Environmental Genetics and Molecular Toxicology, University of Cincinnati College of Medicine, Cincinnati, OH 45267-0056, USA

**Keywords:** semi-synthetic metalworking fluid, machining fluid, microbial diversity, microbial pathogens, Mycobacterium immunogenum, endotoxin, biocide, genus-specific qPCR, PCR-DGGE, amplicon sequencing

## Abstract

Microorganisms colonizing modern water-based metalworking fluids (MWFs) have been implicated in various occupational respiratory health hazards to machinists. An understanding of the exposure risks from specific microbial groups/genera/species (pathogenic or allergenic) and their endotoxins and the need for strategies for effective, timely fluid management warrant real-time extended tracking of the establishment of microbial diversity and the prevailing fluid-related factors. In the current study, the microbial community composition, succession, and dynamics of a freshly recharged industrial semi-synthetic MWF operation was tracked in real-time over a period of 50 weeks, using a combination of microbiological and molecular approaches. Substantial initial bacterial count (both viable and non-viable) even in the freshly recharged MWF pointed to the inefficiency of the dumping, cleaning, and recharge (DCR) process. Subsequent temporal analysis using optimized targeted genus/group-specific qPCR confirmed the presence of Pseudomonads, Enterics, Legionellae, Mycobacteria (*M. immunogenum*), Actinomycetes, and Fungi. In contrast, selective culturing using commercial culture media yielded non-specific isolates and collectively revealed Gram-negative (13 genera representing 19 isolates) and Gram-positive (2 genera representing 6 isolates) bacteria and fungi but not mycobacteria. *Citrobacter* sp. and *Bacillus cereus* represented the most frequent Gram-negative and Gram-positive isolates, respectively, across different media and *Nectria haematococca* isolation as the first evidence of this fungal pathogen colonizing semi-synthetic MWF. Unbiased PCR-DGGE analysis revealed a more diverse whole community composition revealing 22 bacterial phylotypes and their succession. Surges in the endotoxin level coincided with the spikes in Gram-negative bacterial population and biocide additions. Taken together, the results showed that semi-synthetic MWF is conducive for the growth of a highly diverse microbial community including potential bacterial and fungal pathogens, the current DCR practices are inefficient in combating microbial reestablishment, and the practice of periodic biocide additions facilitates the build-up of endotoxins and non-viable bacterial population.

## 1. Introduction

Metalworking fluids (MWFs) are widely used in machining industries for cooling, lubrication, and corrosion prevention during metal grinding and cutting operations. MWF can be divided into four categories, based on their composition: straight oil, soluble oils, synthetic fluids, and semi-synthetic fluids [1,2,3,4]. Of these, semi-synthetic fluids are the most widely used. This fluid type is composed of refined petroleum oils (5–30%), water (30–50%), and emulsifiers, and may be fortified with fatty acids, sulfur, chlorine, and phosphorus derivatives to enhance the performance of the product [2,5]. All these constituents make the semi-synthetic MWF very conducive for sustaining microbial growth, and continuous recirculation of these fluids practiced in the industrial setting leads to a buildup of a diverse microbial community [6,7,8,9] including human pathogens [7,10,11,12,13]. Consequently, microbial colonization of MWF causes deterioration of the fluid’s technical quality and performance [14] as well as ecological and occupational health effects.

Occupational exposure to microbially contaminated MWFs has been associated with various cutaneous and respiratory illnesses such as asthma, hypersensitivity pneumonitis, bacterial pneumonia, among others [15,16,17,18,19,20,21,22,23,24,25,26]. Studies have implied cross-pollination of microbial agents between MWF and human cases of respiratory disease [19,23]. The microbial agents and their products such as antigens and endotoxins released in these fluids have been linked with induction of an inflammatory response in MWF-exposed machine workers [27,28,29,30,31,32,33] and animal models [34,35,36]. The microbial antigens may be contributed by culturable, non-culturable, and non-viable microorganisms. This reinforces the need for monitoring of these fluids for microbial community composition and dynamics, including both culturable and nonculturable microorganisms [8,34,37,38,39].

In previous studies, it has been realized that the traditional culture-based methods used to study microbial contamination levels and diversity in MWF are time-consuming and can detect only <1% of the total bacterial community thereby grossly underestimating the level of contamination [37,40,41,42]. Hence, culture-independent tools such as microscopy or imaging [40,43,44,45] and modern rapid molecular protocols developed in our previous efforts can be used for detection, speciation, and quantitation of the specific microorganisms of all viability statuses prevalent in MWF [4,8,46,47,48,49,50,51,52]. Culture-independent unbiased DNA-based approaches, such as PCR-Denaturing gradient gel electrophoresis (PCR-DGGE), could further reveal the composition and dynamics of the total microbial community [8,39,53,54,55,56]. A combination of molecular and culturing approaches could collectively account for more realistic community composition and dynamics of both culturable and non-culturable populations.

While a majority of the studies cited above have characterized microbial diversity in MWF operations by batch analysis using single or infrequent sampling, little is known about the kinetics of microbial establishment, succession and population dynamics, and the role of fluid-related factors in modulating the microbial community [38,39,57,58]. Particularly, a long-term and frequent tracking of the development of a microbial community in industrial fluid systems could be more informative for understanding and implementing effective fluid management strategies and helping to minimize occupational health hazards from MWF exposures.

In view of the above rationale, the overall objective of this study was to perform an extended real-time tracking of a semi-synthetic in-use metalworking fluid operation that was specially prepared for this investigation using the commonly practiced dumping, cleaning, and recharge (DCR) process, to investigate the process of the establishment of a microbial community in terms of both composition and dynamics. Temporal tracking for over a period of 50 weeks was performed on this operation, which was an integral part of a large automotive-parts manufacturing industrial setting. Specifically, we investigated the following in the MWF operation: (i) total microbial load by culturing versus microscopy (light and epifluorescence); (ii) targeted microbial community composition/structure using a combination of approaches (selective culture, group/genus-specific PCR/qPCR, and amplicon sequencing); (iii) whole microbial community analysis using the unbiased approach PCR-DGGE; and (iv) the endotoxin build-up dynamics. The resulting experimental data were interpreted in light of the information on non-biological fluid parameters periodically recorded and provided by plant management. The results emphasized a high colonization propensity to a broad microbial diversity, a role of certain fluid-related factors in modulating the microbial community and endotoxin build-up, and the importance of temporal tracking using a combination of modern molecular and classical approaches to fully understand the microbial community establishment, dynamics, and diversity in semi-synthetic MWF formulation.

## 2. Materials and Methods

### 2.1. Fluid Sampling

A semi-synthetic in-use metalworking fluid operation located inside a US-based automotive parts-manufacturing plant was investigated in collaboration with the plant’s management. Before initiating sampling for this microbial tracking study, the fluid operation underwent the commonly practiced DCR process to set the baseline. Subsequently, the recharged operation (main pit or reservoir) was pursued over the 50-week sampling period using routine fluid management practices; this included routine periodic “top-loading” (addition of fresh fluid), changing of the fluid, biocide additions, and other practices, as routinely followed in that plant like in other such industrial processes. Two commercial biocides were used, including Kathon-886 MW, a formaldehyde-free biocide and Grotan, a formaldehyde-releasing biocide. The composition of these commercial biocides in terms of active ingredients is as follows: Kathon 886 MW (5-chloro-2-methyl-4-isothiazolin-3-one = 10.4%; 2-methyl-4-isothiazolin-3-one = 3.7%; total active ingredients (typical) = 14.1%), Grotan (hexahydro-1,3,5,-tris (2-hydroxyethyl)-s-triazine = 78.5%). From the day of recharge (“week 0”), the fluid was periodically sampled (one liter sample) from the main pit of the study operation. To characterize the recharging process, we also collected ‘sample before dumping’, ‘neat sample’ (pristine fluid freshly diluted from the same MWF concentrate), and ‘non-circulated sample’ (after recharge but before circulation). The main pit was recharged again (involving the same DCR process) with fresh semi-synthetic fluid at week 17 (before sample SS#18) from the initiation of the tracking study. Another set of ‘neat sample’ and ‘non-circulated sample’ was received along with SS#18 to characterize this second recharging process. Samples SS#1 through SS#25 were drawn consecutively (weekly). There was a two-week-shutdown between samples #25 and #26. Beginning with SS#26, samples were received fortnightly (bi-weekly). In all, 42 samples were drawn over a period of 50 weeks. The samples were designated, as and when received, in the following order: sample before dumping, neat sample #1, non-circulated sample #1, recirculated sample SS#1, SS#2 through SS#17, neat sample #2, non-circulated sample #2, recirculated sample SS#18 through SS# 37. Additionally, the plant’s management provided industrial hygiene information on the MWF operation including dip slide readings (culturable microbial load detected on site) as well as the fluid details such as fresh fluid addition, fluid dilution, fluid pH, oil content, biocide addition, etc. (Supplementary Table S1).

### 2.2. Quantification of Total Microbial Population

An aliquot (20 mL) from each MWF sample was centrifuged (12,000 rpm, 30 min). The resulting microbial pellet was resuspended in 1 mL of normal saline (0.9% sodium chloride solution) and serially diluted before use for microscopy and culturing as follows.

#### 2.2.1. Microscopy

Total bacterial count was determined by light microscopy using Reich slide (Bellco Glass, Inc., Vineland, NJ, USA); this relies on the modified Breed Counting technique [59]. A defined aliquot (5 µL) of the appropriate serial dilution of the microbial cell suspension was smeared in the slide’s marked circle (1 cm^2^ area). Following the standard methylene blue staining (0.3% solution), the smear was examined under the light microscope to determine total bacterial count.

For viable versus non-viable bacterial counts in the sample, epifluorescence microscopy (Nikon Eclipse 50i) was employed using the Baclight Live/Dead viability staining kit (Molecular Probes, Eugene, OR, USA).

For different microscopic counts (total, viable, and nonviable), 10 microscopic fields were screened and the bacterial counts/mL were calculated as follows: Bacterial count (cells/mL) = N_b_ × M_f_ × 1/V × 1/C where N_b_ = average cell number per field, M_f_ (microscopic field) = number of fields per cm^2^ area on the slide, V = volume of stained microbial suspension smeared on the slide, C = concentration factor (fold-concentration of original sample versus the stained sample suspension).

#### 2.2.2. Culturing

Culturable population in the MWF sample was estimated on tryptic soy agar (TSA) by a standard spread agar plating technique using appropriate serial dilutions (100 µL each) of its microbial pellet and incubation at 37 °C for 48 h. The results were calculated as colony forming units (cfu) per ml of the MWF sample.

#### 2.2.3. Viable but Nonculturable (VBNC) Count

The VBMC count in each MWF sample was deduced by subtracting the total culturable count (agar culture-based) from the total viable count (epifluorescence microscopy-based) for that sample.

### 2.3. Targeted Analysis of Microbial Community

#### 2.3.1. Selective Culturing and Identification Using PCR-Amplicon Sequencing (Targeted Community Composition Analysis)

Each MWF sample was cultured for the isolation of specific (targeted) microbial genera/groups (bacterial and fungal) using multiple selective agar media. The isolates thus obtained were identified by bacterial/fungal PCR amplification and amplicon DNA sequencing. Briefly, the microbial cell suspension in saline prepared above for each MWF sample was spread plated (100 µL) using the following genus/group-specific agar media and incubation temperatures: Middlebrook 7H10 agar (MBA; 37 °C + 5% CO_2_) for Mycobacteria, Buffered Charcoal Yeast Extract agar with antibiotics (BCYE; 37 °C + 5% CO_2_) for Legionellae, Pseudomonas Isolation Agar (PIA; 25 °C) for Pseudomonads, Eosin Methylene Blue agar (EMB; 37 °C) for Enteric bacteria, Actinomycetes Isolation Agar (AIA; 30 °C) for Actinomycetes, and Sabourauds Dextrose Agar (SDA; 30 °C) for Fungi. These DIFCO media were obtained from either Difco Labs (Detroit, MI, USA) or Becton, Dickinson & Co. (Sparks, MD, USA).

Visually distinct colony types (bacterial or fungal) were picked from each specific agar medium. Individual bacterial colonies were identified by 16S PCR and amplicon sequencing using primers (Table 1) based on the V2–V3 variable region of the 16S rRNA gene. Likewise, the fungal colonies were identified using PCR-amplicon sequencing based on 18S–28S internal transcribed spacer region. Briefly, the bacterial isolates were identified by culturing in their compatible specific broth and extracting their genomic DNA using our published protocol [60]. The PCR reaction mixture (50 µL) consisted of the following: dNTPs (200 µM); primers (100 ng each); 10× buffer (5 µL); Pfu Ultra high-fidelity DNA polymerase from Stratagene, Cedar Creek, TX, USA (2.5 U); the isolate’s genomic DNA (1 µL). For bacterial PCR, the amplification was performed using an initial denaturation step (95 °C for 2 min); subsequent 30 amplification cycles, each involving 95 °C for 30 s for denaturation, 56 °C for 30 s for annealing, and 68 °C for 1 min for extension; and a final extension step at 68 °C for 5 min. The fungal PCR involved the following amplification conditions: denaturation step (95 °C for 5 min); subsequent 35 amplification cycles, each involving denaturation (95 °C for 1 min); annealing (55 °C for 1 min); and extension (72 °C for 2 min). The amplicons were purified using Millipore size-cut off centrifugation columns and sequenced at the university’s DNA core facility.

#### 2.3.2. Quantitative Real-Time PCR for Specific Genera/Groups (Targeted Community Dynamics)

A real-time quantitative PCR (qPCR) protocol based on group/genus-specific primers (Table 1) was first optimized for the following individual targeted genera/groups: Pseudomonads, Enterics, Mycobacteria, Legionellae, Actinomycetes, and Fungi. Individual standard curves were prepared using the following representative test strains viz. *Pseudomonas fluorescens* ATCC 13525 (Pseudomonads), *E. coli*-DH5α (Enteric bacteria), *Legionella pneumophila* subsp. *pneumophila* ATCC 33215 (Legionellae), *M. immunogenum* ATCC 700506 (Mycobacteria), *Streptomyces griseus* ATCC 11746 (Actinomycetes), and *Phaenerochaete chrysosporium* ATCC 24725 (Fungi). For direct quantification in the fluid sample, genomic DNA was extracted from 100 mL sample aliquot as described earlier (Khan and Yadav, 2004a), followed by real-time PCR amplification on Smart Cycler (Cepheid, Sunnyvale, CA, USA) using Hot start Taq DNA polymerase along with SyBr Green PCR master mix obtained from Stratagene, Cedar Creek, TX, USA, and Takara Bio Inc., Madison, WI, USA. The reaction mixture (20 μL) consisted of 10 μL of 2x master mix added with 40 ng of each primer and 2.5 μL of the template DNA. The common amplification program (with varying annealing temperature) involved initial denaturation at 95 °C/10 min followed by 40 amplification cycles, each using 95 °C/15 s (denaturation), 50–63 °C/15 s (annealing), and 72 °C/30 s (extension). Genus/group-specific annealing temperature used was as follows: 50 °C (Pseudomonads), 60 °C (Enteric bacteria), 57 °C (Legionellae), 58 °C (Mycobacteria), 63 °C (Actinomycetes), and 55 °C (Fungi).

### 2.4. Unbiased (PCR-DGGE) Analysis of Whole Community Composition and Dynamics

Each MWF sample (100 mL aliquot) was centrifuged to isolate total microbial community DNA as described above. The 16S rRNA gene in the V2-V3 region was amplified [66]; the universal primer pair (with a GC-clamp at 5′-end of the forward primer) used had the following sequence: HDA1-GC (5′-CGC CCG GGG CGC GCC CCG GGC GGG GCG GGG GCA CGG GGG GAC TCC TAC GGG AGG CAG T-3′) and HDA2 (5′-GTA TTA CCG CGG CTG GCA C-3′). PCR amplification based on the Ex-Taq DNA polymerase kit (Takara Bio Inc., Madison, WI, USA) was performed using a reaction mixture (50 µL) containing dNTPs (200 μM), primers (100 ng each), microbial DNA (5 µL aliquot), BSA (2.5 µL), and 1.25 U of Taq polymerase. The DGGE analysis of the amplicon mixture was performed on a D-Code Universal Mutation Detection System (Bio-Rad, Hercules, CA, USA). The amplicon mixture obtained was run on a 12% polyacrylamide gel with a 30 to 50% denaturation gradient in 1x TAE electrophoresis buffer. The 100% denaturing solution was comprised of 40% formamide [vol/vol] and 7 M urea. A reference marker was developed in-house based on prominent bands from trial DGGE runs. Electrophoretic separation was performed first at 50 V/15 min followed by 200 V/5 h at 60 °C. Gels stained with ethidium bromide (0.5 µg/mL for 10 min) were imaged using Kodak Gel Documentation 1D image analysis software version 3.5 (Rochester, NY, USA). DICE/UPGMA Clustering and analysis for the banding patterns corresponding to the individual samples were performed using GelCompar II software version 4.5 (Applied Math, Keistraat, Belgium).

### 2.5. Endotoxin Analysis in MWF

All MWF samples were analyzed for endotoxin level based on Chromogenic Limulus Amebocyte Lysate (LAL) assay using a commercial kit (Cambrex, Walkersville, MD, USA). For this analysis, the sample supernatants obtained above from the microbial pelleting step by centrifugation (12,000 rpm for 30 min) were serially diluted using endotoxin-free water. The diluted sample and the LAL reagent were mixed in equal volumes (50 µL each) followed by incubation (37 °C for 10 min). An aliquot of chromogenic substrate (100 µL) was then added followed by incubation (6 min). After stopping the reaction with 25% acetic acid, absorbance (405 nm) was measured using a Wallac Victor2 plate reader (Perkin Elmer, Boston, MA, USA). A standard curve was generated using the kit’s endotoxin standard (Cambrex, Walkersville, MD, USA) for deducing the endotoxin concentration in the samples.

## 3. Results and Discussion

Over the years, it has been recognized that significant health and safety risks exist for machine workers exposed to microbially contaminated MWFs [18,19,20,21,22,23,24,25,26,67,68,69,70,71]. In terms of the etiology of health effects, while the infectivity of MWF microbes may be dependent on viability, the immunogenicity is independent of their viability status as non-viable cells may carry the causal antigens repertoire; hence, there is a need to determine the load of both viable as well as non-viable populations of the microbial community in the metalworking fluid.

### 3.1. Microbial Population Dynamics (Viable versus Non-Viable)

**Figure 1:**
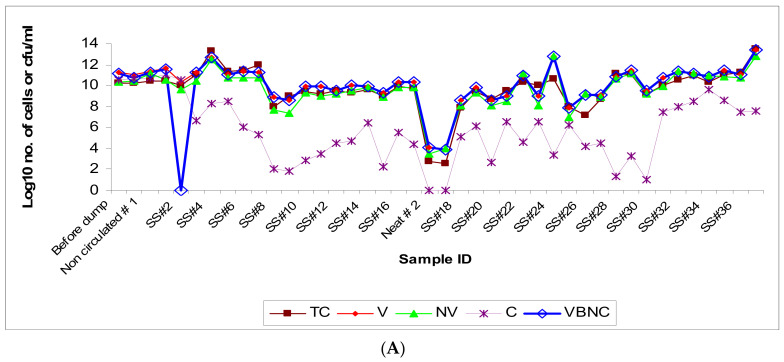

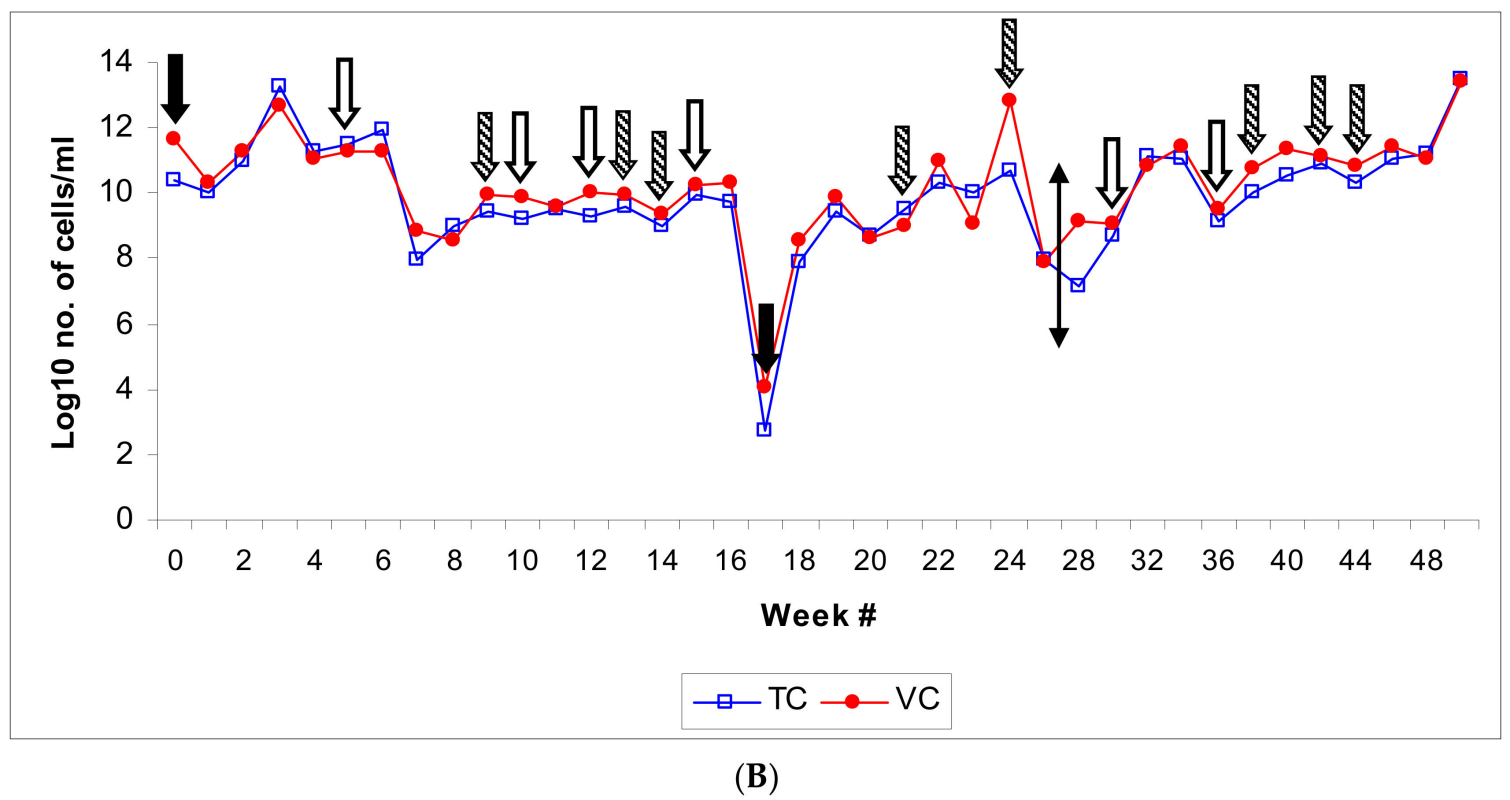
(**A**): Population dynamic changes in the microbial population in a semi-synthetic metalworking fluid (MWF) operation tracked for 50 weeks based on microscopic and culturable counts on the temporally-drawn MWF samples. The sampling schedule is detailed in the Section 2. Abbreviations: TC (total count); V (viable count); NV (nonviable count); C (culturable count); VBNC (viable but nonculturable count). The bacterial numbers are expressed as microscopic count (TC, V, NV, and VBNC) or cfu (C) per ml of the fluid. (**B**): Microbial population dynamic changes (total and viable bacterial counts) in relation to changes in fluid parameters in the semi-synthetic MWF operation. The samples were drawn from the semi-synthetic MWF operation over a 50-week period during which time it was “top-loaded” or “recharged” with fresh fluid, or treated with biocides at different time intervals. The fluid sampling schedule is described under Section 2 and the fluid’s physical/chemical parameters are described in Supplementary Table S1. The figure indicates- recharge of the pit/reservoir with semi-synthetic MWF fluid (solid arrow), top-loading of the pit/reservoir with semi-synthetic MWF fluid (striped arrow), and addition of biocides Kathon or Grotan (blank arrow). The two-headed arrow indicates a 2-week temporary shut-down of the operation after week 25.

Microscopic quantification after methylene blue staining revealed high levels of total bacterial counts in the ‘sample-before dumping’ and substantial microbial load even in the initial baseline samples including the ‘neat’ sample (made up in-house at the plant) and the ‘non-circulated’ sample. This indicates that the DCR (dump, clean, recharge) process was inefficient in decontamination of the fluid pit/reservoir. The residual microflora, potentially from biofilms [6,8,72,73] commonly resistant to regular cleaning/disinfection and persistent on the interior surfaces in the pipes and at the bottom in fluid reservoirs, might have contributed to the high baseline microbial load in these initial samples while the freshly added fluid provided nutrients for the growth of microorganisms in the recharged pit [57,74]. The total microbial population remained at the same level in the initial three samples (SS#1–3) and showed a slight increase by 1-log value in the next four samples followed by a decline by 2–4 log values in the subsequent samples (SS#8 through 17) received from the first recharge process (Figure 1A). VBNC decline during SS#1–3 may likely be due to a lag in manifestation of the fresh fluid’s built-in biocidal activity. The main pit was recharged the second time at week 17, and the population count in the ‘neat’ sample and the ‘non-circulated’ sample collected during this second recharging process was found to be ~10^2^ cells/mL, showing the presence of a residual/baseline microbial community in the freshly “cleaned/recharged” process. The bacterial population showed a sudden increase by 5-log values in just one week since the recharging (as reflected in sample #18 at week 18). The samples received from this second recharge process showed frequent fluctuations but remained at high levels, falling in the range of 10^7^–10^11^ cells/mL, and reached the highest count in the last sample, SS#37 (Figure 1A). The high total bacterial counts detected in the present study were consistent with those reported previously from the water-based MWF matrices [4,11,39,43,75,76]. Although the residual/baseline microbial flora (the seeding flora) after the second DCR was at much lower level than that observed after the first DCR, the total microbial population quickly reached the same level in the two post-DCR scenarios, implying a critical role of fluid factors in the establishment and dynamics of the microbial population.

Population dynamic changes in total bacterial community appeared to be a result of the corresponding increases in viable subpopulation and the non-viable subpopulation. The observed slow rate of increase in the viable subpopulation in the initial 6-week period after the first DCR could be due to the adaptation of MWF microflora to the new fluid. This is in contrast with the post-second DCR scenario where the population rebounded to the original levels fairly rapidly likely because of pre-adaptation of the residual/seeding microflora as the same fluid brand was used for recharging. The viable but nonculturable (VBNC) fraction increased in parallel with the total viable count indicating bacteriostatic effect of the in-use MWF despite periodic biocide additions. A majority of the population was non-culturable indicating the loss of cultivability due to the largely bacteriostatic environment in the MWF operation. The changes in the culturable fraction of the population coincided with the fresh fluid addition (showing an increase, particularly in advanced stages of the fluid operation such as at weeks 9 and 14) and biocide addition (showing a decrease, as observed at weeks 5, 15, and 30). The increase in non-viable subpopulation could be attributed to the short lifespan of bacteria (spontaneous death) and the bactericidal effect of the added biocides (Kathon and Grotan) on certain microbial genera in the test fluid operation. Differential efficacy of the two biocides used in this operation, Kathon and Grotan, towards the prevalent microbial genera Pseudomonas and Mycobacterium in semi-synthetic MWF has been reported in our earlier published in vitro simulation study [77]. The highly fluctuating levels of total microbial population such as those during the initial period following the second recharge process (at week 17) might be due to frequent fluid additions (at week 21 and 24) (Figure 1B). Biocide addition did not cause an immediate decrease in the total population levels likely because of the acquired biocide resistance in the established microbial species and/or differential efficacy of the used biocides (Kathon and Grotan) towards certain fractions of the population under the prevailing fluid microenvironment, as demonstrated in our in vitro simulation experiments with these biocides [77]. Interestingly, the addition of biocide Kathon (but not Grotan) was associated with an increase in total/tramp oil content, which coincided with an increase in microbial population as observed in SS#28 (Kathon added at week 30 in SS#27) and SS#31 (Kathon added at week 36 in SS#30) (Figure 1B, Supplementary Table S1).

### 3.2. Microbial Community Diversity (Composition and Dynamics)

#### 3.2.1. Targeted Community Profile

##### Selective Culturing and DNA Sequencing

**Figure 2:**
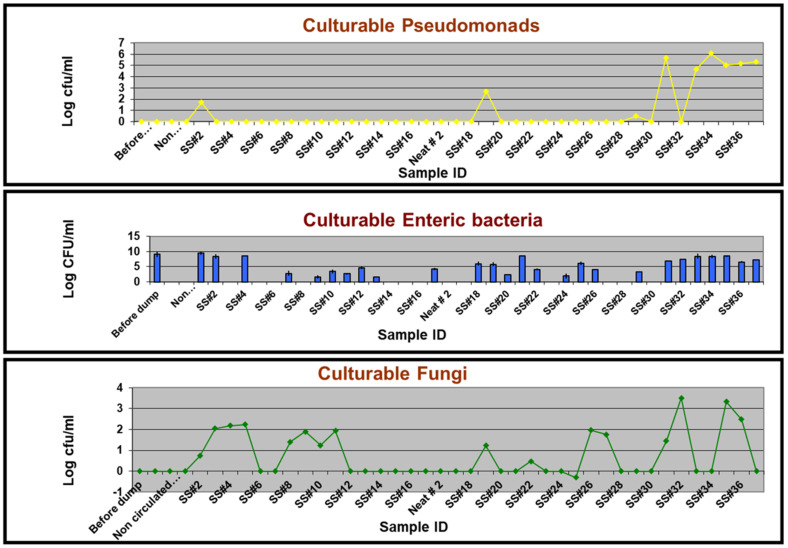
Selective culture-based quantification of the targeted groups in temporally drawn MWF samples. The quantitative levels of a test group/genus are expressed as CFU per ml of the fluid, determined as described in the Section 2.

Six different genera isolated on a non-specific/general medium (TSA) were identified by DNA sequencing as *Alcaligenes*, *Caulobacter*, *Citrobacter*, *Methylobacterium*, *Pseudomonas,* and *Staphylococcus* (Table 2). The six commercial selective media originally designed for isolating the targeted bacterial genera/groups either yielded the targeted group along with non-targeted genera/groups or did not yield the intended target genus/group (Table 2). For instance, PIA medium grew Enterics and other Gram-negative bacteria in addition to Pseudomonads. Similarly, EMB grew other Gram-negative and Gram-positive bacteria in addition to Enterics. However, MBA, BCYE, and AIA did not yield any isolate of the targeted genera/groups, namely, Mycobacteria, Legionellae, and Actinomycetes, respectively, but grew other bacterial and fungal colonies. This revealed that these presumably selective media from commercial sources are not absolutely selective. Even when there was relevant/specific detection of the targeted group, the selective culturing approach yielded a positive outcome for only certain samples (Figure 2). As an example, PIA culturing did not yield any colonies from any of the samples following the first recharge process (samples 1 through 17) except for SS#2. Instead, fungal isolates were obtained on this agar from SS#3, 4, 5, 19, and 27. These fungal isolates were identified by ITS amplicon sequencing as *Nectria haematococca*. Subsequently, typical Pseudomonads colonies were obtained on this medium from samples #19 and 29, albeit at low levels and from samples #31 and #33 through #37 at levels varying from 10^4^ to 10^7^ cfu/mL. EMB did not yield enteric bacteria from the neat and the non-circulated samples drawn after the two recharged processes and instead grew *Bacillus cereus* from non-circulated sample #1. However, an enteric population was detected in other samples except SS#3, 5, 6, 8, 14, 15, 16, 23, 27, 28, and 30, at levels varying from 37.5 cfu/mL to 2.9 × 10^8^ cfu/mL, in addition to other Gram-negative and Gram-positive bacterial species (Table 2). MBA did not yield any Mycobacteria from any of the samples but grew *B. cereus* and *Alcaligenes* sp. from non-circulated sample #1 and SS#31, respectively. Similarly, no culturable Legionellae and Actinomycetes were isolated from any of the samples on the respective specific media BCYE and AIA. SDA meant for the growth of fungi did not yield any fungal colonies from the first three DCR samples (before dump, neat, and non-circulated) and SS#1. However, fungal population was detected in the subsequent samples except SS#6, 7, 12, 13, 14, 17, 18, 21, 23, 24, 28, 29, 31, 33, 34, and 37 at levels varying from 1 to 3.25 × 10^3^ cfu/mL. The typical fungal colonies were identified as *N. haematococca* based on ITS sequencing.

Overall, the results from selective culturing revealed that the microbial community of the test fluid consisted of a mixture of Gram-positive and Gram-negative bacteria and fungi. The bacterial genera cultured from the MWF samples in our study have been previously reported from various MWF matrices [11,15,37,56,58,76,78,79,80], but the current study contrasts in terms of delineating their temporal establishment and dynamics in the fluid and role of the underlying fluid factors in this context. *Citrobacter* sp. was the most frequent bacterial isolate across different media (four of the seven media used), indicating its versatile cultivability. Detection of Citrobacter is significant considering that this genus has several pathogenic species, which can cause urogenital, gastrointestinal, and various systemic infections in humans and is considered a growing threat to public health [81]. Our findings on fungal colonization contrasted with an earlier suggestion that a permanent or temporary die-off of pioneering bacterial colonizers results in colonization with fungal organisms [82] but are consistent with subsequent studies that reported the simultaneous presence of both bacteria and fungi [6,7,8,9]. *N. haematococca*, a known phytopathogen recently implicated in human keratitis cases [83], was the only fungal isolate obtained from the test fluid operation. To our knowledge, this fungal species has not been reported from semi-synthetic MWF. Common fungal contaminants reported in MWF matrices are species of *Penicillium*, *Fusarium*, *Aspergillus*, and *Cladosporium* sp. [7,39,56,58].

##### Real-Time qPCR and Amplicon Sequencing

**Figure 3:**
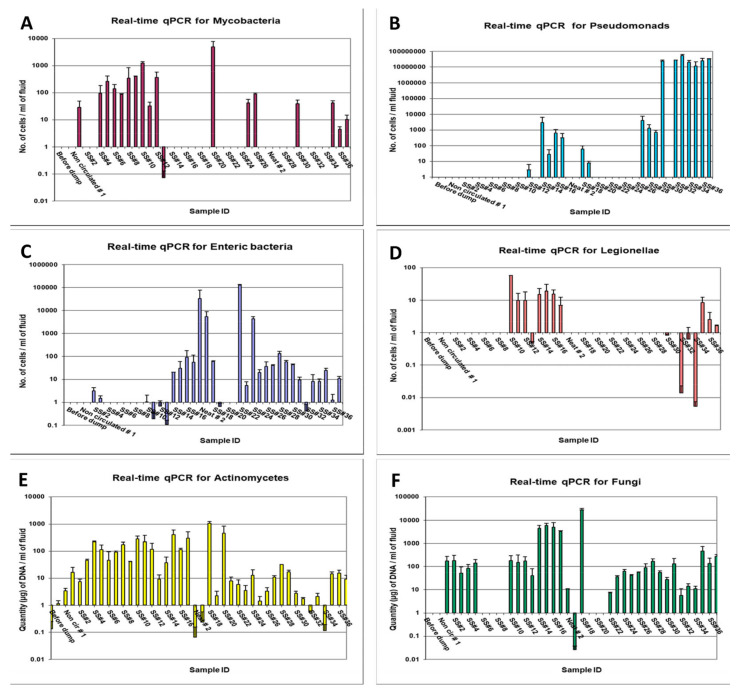
Real-time qPCR-based quantification of the targeted groups/genera in temporally drawn MWF samples. The quantitative levels of a test group/genus are expressed as counts per ml (**A**–**D**) or microgram DNA per ml (**E**,**F**) of the fluid, calculated as described in the Section 2.

Mycobacteria were not detected in the three DCR samples (before dump, neat, non-circulated). However, mycobacteria were detected in the first recirculated sample (SS#1) at a level of 2.18 × 10^1^ cells/mL but were again non-detectable in subsequent samples, SS#2 and 3 (Figure 3A). The mycobacterial number showed a gradual increase from 8.28 × 10^1^ cells/mL in sample #4 to 1.64 × 10^3^ cells/mL in SS#13. Thereafter, mycobacteria were not detected in any of the samples, except sample #20 and 35, at a level of 4.6 × 10^3^ cells/mL and 4.16 × 10^1^ cells/mL, respectively, and in SS#36 and 37 at levels below the minimal detection limit. Molecular speciation of *Mycobacterium* from these samples (samples SS#6 through 13), performed using DNA sequencing and BLAST homology search on *hsp* gene amplicon, revealed nearly 100% homology (maximum identity = 99–100%, maximum score = 414–420) with *M. immunogenum*, which is the species most frequently isolated from mycobacteria-contaminated MWFs [10,17,84,85,86].

Pseudomonads were not detected in two of the first DCR samples (sample-before dump and neat sample) but were detected in the non-circulated sample and SS#2, albeit at very low levels (6.9 cells/mL and 9.39 cells/mL, respectively). Subsequently, Pseudomonads were detected in SS#13 through 19 at a level varying from 1.42 × 10^0^ to 1.62 × 10^3^ cells/mL and in SS#25 through 31, except SS#26, at a level varying from 4.14 × 10^1^ to 3.3 × 10^3^ cells/mL, indicating their gradual multiplication in the fluid during this sampling range. The Pseudomonads population, however, increased by 5-log values in SS#32 through 37, where the counts varied from 1.02 × 10^7^ to 3.48 × 10^7^ cells/mL (Figure 3B).

Enteric bacteria were not detected in any of the samples following the first recharge process (SS#1 through 17), except in SS#2, 3, 10, 12, and 14 through 17 at levels varying from 1.46 to 66 cells/mL. However, enteric bacteria were detected in the neat, non-circulated, and the recirculated sample SS#18 from the second recharge process at 4.3 × 10^4^ cells/mL, 3.6 × 10^3^ cells/mL and 60 cells/mL, respectively. Subsequently, enteric bacteria were detected in SS#25 through 37 at 10–100 cells/mL in all the samples except SS#32 and 35 through 37 where they were detected at levels lower than the minimal detection limit (Figure 3C).

Legionellae were detected in only SS#10 through 17 from the first recharge process at levels varying from 5.8 to 57.8 cells/mL and in SS#30 and 32 through 37 from the second recharge process at levels below the minimal detection limit (Figure 3D).

Actinomycetes were detected in all the first DCR samples (sample-before dump, neat sample, and non-circulated sample at levels 0.13, 1.15, and 3.43 µg DNA/mL, respectively). However, the actinomycetes population did not show any clear trend and was detected at levels varying from 7.35 µg DNA/mL to 297.55 µg DNA/mL in SS#1 through 17. The neat and non-circulated samples from the second recharge process had very low levels of actinomycetes populations (0.06 and 0.23 µg DNA/mL). However, the actinomycetes population increased drastically in SS#18 (1074.16 µg DNA/mL) but again declined in SS#19. Subsequently, actinomycetes were detected at widely varying levels ranging from 0.11 to 31.52 µg DNA/mL in samples #21 through 37 (Figure 3E).

Fungal population was not detected in the initial DCR samples (sample collected before dumping, the neat sample, and the non-circulated sample). However, fungi were detected in recirculated sample SS#1 and 2 at 171.36 and 182.28 µg DNA/mL. The population declined by 2–3-fold in the next two samples but returned to the same level in SS#5 and in samples #10 through 12, with an intermediate absence in samples #14–17. The neat and non-circulated samples from the second recharge process had fungal populations at the levels 10.25 and 0.026 µg DNA/mL, respectively. However, the fungal population was detected at an extremely high level in recirculated sample #18, followed by an absence in samples #19 through 21. Thereafter, fungal population was detected at levels varying from 5.74 to 475.8 µg DNA/mL in samples #22 through 37 (Figure 3F).

Taken together, the results of real-time qPCR showed the presence of all the targeted microbial genera/groups in the semi-synthetic fluid samples; however, the population dynamics for the individual targeted genera/groups did not follow any specific trend, which may possibly be due to frequent fluid additions in the form of top-loading and recharging (DCR) of the pit and periodic biocide additions. Actinomycetes, Mycobacteria, and Legionellae were not detected by culture in any of the samples but were detected by qPCR in some or all the samples indicating that most of the target groups/genera are present in non-culturable form. Among the specific targeted groups analyzed based on qPCR analysis, Pseudomonads were found to be the predominant group of microorganisms, as has been reported in earlier studies using culturing or genotypic analysis [6,9,38,56,58,76]. Actinomycetes and mycobacteria have been associated with the etiology of occupational hypersensitivity pneumonitis (HP) in epidemiological studies on farmers and machinists [58,87]. Since HP is an immune-mediated disease, even non-viable cells of these organisms can induce the disease. In this context, rodent studies including our own using cell lysates have demonstrated immunogenicity and role of preformed antigens of these organisms in inducing HP-like lung pathology [36].

### 3.3. Whole Community Composition and Succession (Based on DGGE Analysis)

**Figure 4:**
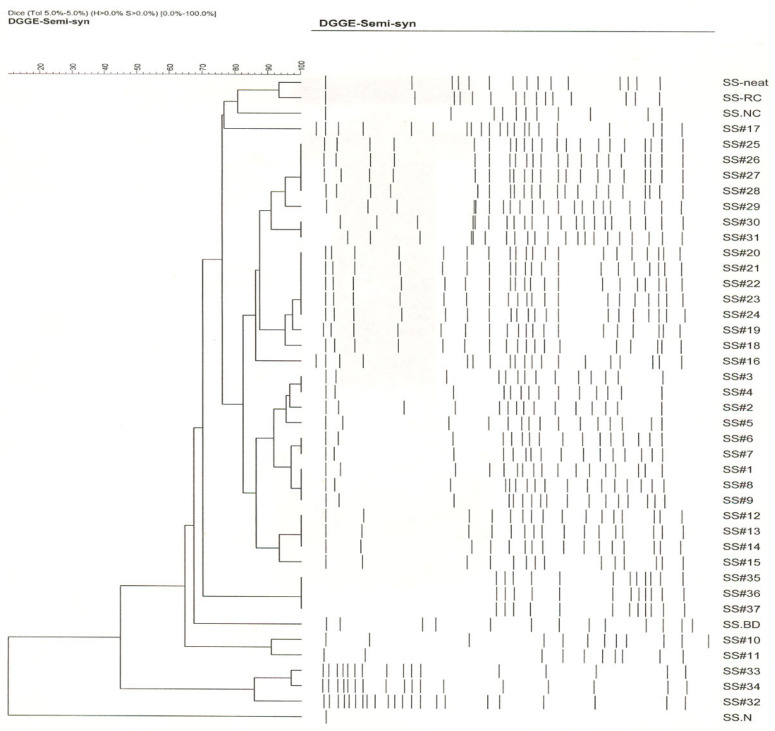
Global microbial community composition and dynamics as revealed by Dice/UPGMA clustering of DGGE fingerprints of semi-synthetic MWF samples. The samples were grouped into 8 clusters. The band tolerance of 5% was used.

DGGE analysis of the 16S rRNA gene amplicons of the 42 semi-synthetic samples showed the presence of 10–22 different bacterial types. Based on the banding patterns in GelCompar analysis, the 42 samples could be grouped into 8 clusters (Figure 4). SS#1 from the first recharge process showed a banding pattern comprised of 17 bacterial types and differed from the DCR samples-before dump and non-circulated—by 22% and 14.5%, respectively, indicating a major difference in the community composition from these initial samples. SS#2 showed a banding pattern consisting of 15 bacterial types and differed from that of SS#1 by 2%. SS#3 and 4 showed the same banding pattern and were therefore grouped in cluster 1 and differed from SS#2 and 5 by 4% and 5%, respectively. Samples SS#6 and 7 were grouped into cluster 2 and differed from cluster 1 by 4%. Similarly, samples SS#8 and 9 were grouped into cluster 3 and differed from cluster 2 by 4%. Samples SS#10 and 11 showed a banding pattern differing from cluster 3 by 6% each. Samples SS#12 through 14 were grouped into cluster 4 and differed from SS#11 by 2% and SS#15 by 8%. SS#16 and 17 showed distinct banding patterns differing from one other by 8.5%. SS#18, the first recirculated sample from the second recharge process showed a major community shift and a banding pattern consisting of 18 bands, which differed from that of SS#17 (last sample from the previous recharge process) by 16.5%. SS#19 showed a banding pattern differing from that of SS#18 by 2%. Samples SS#20 through 24 were grouped into cluster 5 while samples SS#25 through 28 were grouped into cluster 6 and differed from each other by 2%. SS#30 and 31 were grouped into cluster 7 and differed from SS#29 and 32 by 4.5% and 6.5%, respectively. SS#33 showed a distinct pattern differing from SS#32 and 34 by 10% and 4%, respectively. Samples #35 through 37 showed an identical pattern consisting of 12 bacterial types and were grouped into cluster 8.

The DGGE results implied prevalence of a broader microbial community diversity in the tracked industrial semi-synthetic fluid system. This contrasts with the earlier findings [56,58,76,88], where the microbial communities in industrial spent MWFs analyzed using phenotypic and genotypic methods showed low diversity. Overall, the microbial community composition of the samples from the first recharge process (samples SS#1 through 17) varied from one another by 2% to 8.5%. There was a major drift (as the banding pattern of SS#18 from the second recharge process differed from that of SS#17, the last sample from the first recharge process, by 16.5%) in the microbial community after the second recharge process, and the samples from this recharge process (samples #18 through 37) varied from one another by 2 to 10%. Many (8/17 from first recharge and 6/20 in second recharge process) of the samples from both the recharge processes showed a distinct banding pattern and were therefore not groupable into clusters, indicating frequent shifts in microbial community possibly caused by frequent fluid additions and relative effects of biocide replenishments.

### 3.4. Endotoxin Build-Up and Dynamics

**Figure 5:**
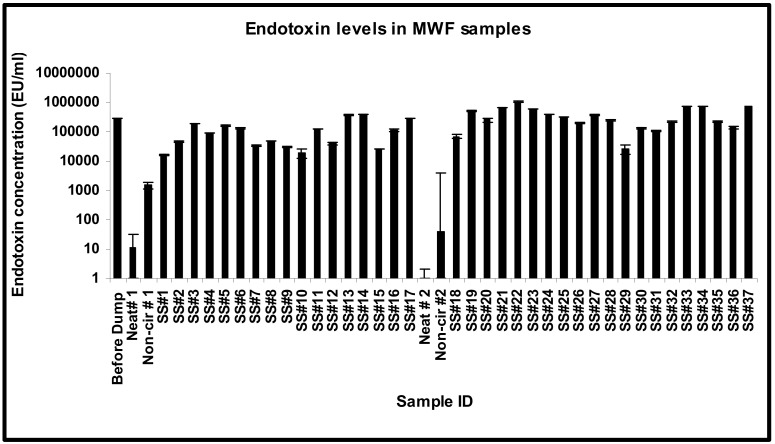
**Endotoxin concentration pattern in temporally drawn MWF samples.** The sampling schedule spanned a period of 50 weeks as described in the Section 2.

The endotoxin levels in the MWF samples were highly variable, ranging from non-detectable to 1 × 10^6^ EU/mL, which was close to that detected in other studies [89]. The endotoxin level was very high (300,036 EU/mL) in the sample collected before dumping and was very low in the neat sample (11.46 EU/mL). The endotoxin levels showed a sharp increase from 1546.9 EU/mL in the non-circulated sample to 181,984 EU/mL in SS#3, showed a slight decline in SS#4, remained almost at the same levels in SS#5 and 6, and then showed a gradual decline in samples SS#7 through 9 (Figure 5). The high endotoxin levels correlated with the high total microbial population in sample collected before dumping but not in the neat sample. The increase in endotoxin levels from the non-circulated sample through SS#3 did not correlate with the total microbial population and thus could be the result of the rinsing of residual endotoxin from the surfaces/crevices. However, the decline in the endotoxin levels from samples SS#7 (week 6) through 9 (week 8) coincided with the decline in the total microbial population in these samples. There was a sudden surge in the endotoxin concentrations in SS#11 (week 10), 13 (week 12), and 14 (week 13) (126,602 EU/mL, 368,377.7 EU/mL, and 408,059.9 EU/mL, respectively) in comparison to SS#10 (week 9) and 12 (week 11), which had endotoxin levels of 19,397.48 EU/mL and 41,144.43 EU/mL, respectively. The surge in SS#13 coincided with biocide addition at week 12. SS#15 showed a decrease in endotoxin levels (25,812.51 EU/mL) followed by a surge in SS#16 (week 15) and 17 (week 16), which coincided with biocide addition at week 15. The pit was recharged (second DCR) after this sample was drawn. Analysis of the fresh fluid (neat) and non-circulated sample during this second DCR process showed that the endotoxin was undetectable in the neat sample and was present at a very low level (39.42 EU/mL) in the non-circulated sample (Figure 5), indicating that the major source of endotoxin is the multiplying bacterial population and not the system or the biofilms. Thereafter, a steady level of endotoxin was observed in SS#19 through 28, with a minor drop in levels in SS#26 and 28. However, this fluctuation was relatively negligible in comparison to SS#29 that showed a 10-fold decrease in endotoxin concentration. This dip corresponded to a decrease in the Gram-negative bacterial population from SS#28 to 29, as measured in terms of Enterics and Pseudomonads. This correlation between levels of Gram-negative population and endotoxins is consistent with earlier reports [90,91,92]. There was a 5-fold increase in the endotoxin levels in SS#30, coinciding with biocide addition, and the same level continued in SS#31. There was a subsequent 2-fold increase in SS#32, and then a further 3-fold increase in samples #33 and 34. This increase coincided with an increase in the population of Gram-negative bacteria, particularly Pseudomonads, from 10^2^ cells /mL in SS# 31 to 10^7^ cells/mL in the subsequent samples (SS#32 through 37). There was, however, an unexpected decline in the endotoxin levels in samples #35 and 36, but endotoxin concentration in SS#37 returned to the same levels as SS#34.

The changes in the endotoxin levels coincided with the additions of fresh fluid such as at week 9, 14, 21, and 34 or biocide addition such as at week 12, 15, and 36 (Supplementary Table S1), possibly due to enhanced multiplication (fresh fluid-caused) and/or lysis (biocide-caused) of Gram-negative bacteria. Taken together, endotoxin levels may serve as a surrogate marker to assess the Gram-negative bacterial population burden in the fluid. Such endotoxin-based assessment of the burden of Gram-negative organisms such as Pseudomonas as well as high endotoxin levels may be significant in the assessment of machine workers’ risk of occupational lung disease. In this context, several studies have associated lung inflammatory response in machine workers with exposure to microbial endotoxins and antigens in the machining fluid [27,28,29,30,31,32,33]. Initial studies on the etiology of hypersensitivity pneumonitis (HP) in machinists implicated Pseudomonas as the etiological agent [67], and later studies from our laboratory have shown exacerbating effects of Pseudomonas in MWF mycobacteria-induced HP-like pathology in mice (unpublished). Other MWF mycobacteria-induced HP mouse model studies have shown confounding effects of endotoxin [35].

## 4. Study Limitations and Future Directions

It is significant that the current study enabled investigation of the temporal dynamics of microbial colonization and succession vis-à-vis various fluid-related factors. However, further parallel information on the dynamics of fluid factors such as temperature fluctuations, biocide concentration changes, etc., could have been useful in further interpretation of the data. The current study design employed a combination of culture and DNA-based techniques including targeted amplicon DNA sequencing. Future studies may be designed using a global high throughput nextgen sequencing of amplicons to generate more in-depth information on microbial diversity. In terms of follow up studies on treatment effects in such MWF operations, the DCR process may be monitored more closely so the seeding of fresh fluid with the residual microbial contaminants can be minimized. Appropriate real-time monitoring for the prevailing microbial diversity in the in-use fluid may guide the selection of an appropriate biocide brand to achieve higher efficacy in microbial control thereby minimizing fluid deterioration and occupational health risks.

## 5. Conclusions

The study findings indicated that semi-synthetic MWF formulation has the propensity for colonization by and growth of a highly diverse microbial community comprised of Gram-negative bacteria, Gram-positive bacteria, acid-fast bacteria, and fungal organisms, including potentially pathogenic genera/groups (Mycobacteria, Legionellae, Enterics, Pseudomonads, Nectria). PCR analysis showed Pseudomonads as the most dominant bacterial contaminant group. DNA-based analysis revealed contamination with mycobacteria (*M. immunogenum*) and Legionellae, but the selective culturing efforts did not yield any isolates, implying non-viable or viable but nonculturable populations of these respiratory pathogens. The pathogenic fungus *Nectria haematococca* was the only culturable fungal species detected in the fluid samples. The endotoxin build-up correlated with the Gram-negative bacterial population dynamics. The results revealed inadequacy of the DCR process in eliminating microbial reestablishment in the fluid operation. Fluid-related factors, particularly fresh fluid ‘top-loading’ and biocide addition, facilitated modulation of the microbial community and endotoxin build-up (via induction of nonviable bacterial population), respectively. Collectively, these observations emphasize the importance of improving the DCR process and periodic tracking of the fluid colonization based on a combination of modern molecular and classical approaches to fully understand and timely intervene in the process of microbial community establishment and progression in industrial semi-synthetic MWF operations.

## Figures and Tables

**Table 1:** List of group/genus-specific primers used for real-time qPCR in the current study.

Targeted Group/Genus	Primer Sequence	Source Reference
Pseudomonads	Forward-5′ GAG TTT GAT CCT GGC TCA G 3′ Reverse-5′ CCT TCC TCC CAA CTT 3′	[61]
Enteric Bacteria	Forward-5′ TCC GTG AAA AGG GCA CTA AC 3′ Reverse-5′ CAG ATC GGA CAT CAA ATA GC 3′	[62]
Mycobacteria	Forward-5′ CTG GTC AAG GAA GGT CTG GC 3′ Reverse-5′ GAT GAC ACC CYTC GTT GCC AAC 3′	[47]
Legionellae	Forward-5′ AGG GTT GAT AGG TTA AGA GC 3′ Reverse-5′ CCA ACA GCT AGT TGA CAT CG 3′	[63]
Actinomycetes	Forward-5′ GGA TGA GCC CGC GGC CTA 3′ Reverse-5′ CGG CCG CGG CTG CTG GCA CGT A 3′	[64]
Fungi	Forward-5′ GGC TCT CGC ATC GAT GAA GAA C 3′ Reverse-5′ CTT TTC CTC CGC TTA TTG ATA TGC 3′	[65]

**Table 2:** Microbial isolates cultured from MWF samples using general or selective culture media.

Culture Medium ^¥^	Isolate(s)
TSA	*Alcaligenes* sp., *Caulobacter* sp., *Citrobacter* sp., *Methylobacterium* sp., *Pseudomonas* sp., *Staphylococcus* sp. *
EMB	*Acidovorax* sp., *Alcaligenes* sp., *Bacillus cereus* *, *Citrobacter* sp., *Morganella morganii*, *Pseudomonas* sp., *Sinorhizobium* sp.
PIA	*Achromobacter* sp., *Aeromonas* sp., *Citrobacter* sp., *Morganella morganii*, *Serratia* sp., *Stenotrophomonas* sp.
MBA	*Alcaligenes* sp., *Bacillus cereus* *
BCYE	*Sinorhozobium* sp., *Sphignobacterium* sp.
AIA	*Bacillus cereus* *, *Citrobacter* sp., *Nectria haematococca* ^#^
SDA	*Enterobacter* sp., *Nectria haematococca* ^#^

^¥^ Full names of the general (TSA) and selective culture media (other media) are described in Section 2. The media compositions were obtained from manuals on standard microbiological media * Gram-positive isolates (Gram-negative isolates are unmarked). ^#^ Fungal isolates.

## Data Availability

Data from this study are available from the corresponding author upon reasonable request.

## Supplementary Materials

The following supporting information can be downloaded at: https://www.mdpi.com/article/10.3390/microorganisms12020267/s1. Table S1: Non-biological information on the samples from industrial Semi-Synthetic metalworking fluid operation.
