# Supplementary material for: Microbial Community Establishment, Succession, and Temporal Dynamics in an Industrial Semi-Synthetic Metalworking Fluid Operation: A 50-Week Real-Time Tracking
Source: Microorganisms. 2024 Jan 26;12(2):267. doi: 10.3390/microorganisms12020267 (PMC10891577; doi:10.3390/microorganisms12020267)
Supplement: Supplementary file 1 [file microorganisms-12-00267-s001.zip › microorganisms-2838471-supplementary.pdf]

Supplementary Table S1. Non-biological information on the samples from industrial Semi-Synthetic metalworking fluid operation

Sample No.	Fluid details					Biocide addition		Oil content		Dip slide (Biostrip)
	New additions	Final dilution (%)	pH	Ammonia odor #	Amine (%)	Type	Conc.	Tramp oil	Total oil	
Before Dump	NA	NA	NA	NA	NA	NA	NA	NA	NA	NA
Neat (Sample made-up in the lab) # 1		NA	NA	NA	NA	NA	NA	NA	NA	NA
Non-circulated # 1	Klear Kool 3089 (Fresh addition/recharge)	NA	NA	NA	NA	NA	NA	NA	NA	NA
Recirculated/SS1 (wk 00)	-	NA	NA	NA	NA	-	-	NA	NA	NA
SS2 (wk 01)	-	10	8.7	none	NA	-	-	1	1.2	10^3
SS3 (wk 02)	-	10	9.46	none	NA	-	-	3.0	7.0	10^5
SS4 (wk 03)	-	12	9.26	none	NA	-	-	4.5	8.6	10^2
SS5 (wk 04)	-	8.5	8.97	none	NA	-	-	3.5	8	10^3
SS6 (wk 05)	-	12	9.28	none	NA	1000g Kathon 893	NA	3.5	8	Mold presence
SS7 (wk 06)	-	9	9.08	none	NA	-	-	5	10	NA
SS8 (wk 07)	3 gal Amp 95	10	9.25	none	NA	-	-	6.5	10	10^3
SS9 (wk 08)	-	9	9.15	none	NA	-		7.0	10	0
SS10 (wk 09)	10 gal Klear Cool	11	9.10	none	NA	-	-	5.0	10	0
SS11 (wk 10)	-	9	9.17	none	NA	1000g Kathon 893	NA	2.5	7.0	10^3
SS12 (wk 11)	-	9	8.95	NA	NA	-	-	3.0	5.7	0
SS13 (wk 12)	-	9	8.86	none	NA	500 g Kathon	NA	4.0	7.8	NA

						893				
SS14 (wk 13)	33 gal Klear Cool	8.5	8.80	none	NA	-	-	2.0	5.7	NA
SS15 (wk 14)	33 gal Klear Cool	8.5	8.87	none	NA	-	-	2.0	6.0	NA
SS16 (wk 15)	-	11	9.16	none	NA	3 gal Grotan	NA	1.5	5.5	10^5
SS17 (wk 16)	-	8.5	8.89	none	NA	-	-	1.5	4.0	10^3
Neat # 1	Klear Cool	9.0	9.6	none	NA	-	-	0	2.0	0
Non –circulated # 2	Fresh recharge of 140 gallons of Klear Kool 3089	9.0	10.0	none	NA	-	-	0	2.0	0
SS18 (wk 18)	-	9	9.60	none	NA	-	-	0	2.0	0
SS19 (wk 19)	-	11	8.65	none	NA	-	-	1.0	5.0	0
SS20 (wk 20)	-	11	8.97	none	NA	-	-	1.0	4.0	10^3
SS21 (wk 21)	40 gal Klear Cool	4.5	9.13	none	NA	-	-	1.0	4.0	10^3
SS22 (wk 22)	NA	NA	NA	none	NA	-	-	NA	NA	NA
SS23 (wk 23)	NA	NA	NA	none	NA	-	-	NA	NA	NA
SS24 (wk 24)	30 gal Klear Cool	11.0	8.9	none	NA	-	-	0.5	7.0	10^3
SS25 (wk 25)	-	10.0	9.0	none	NA	-	-	0.5	6.5	10^3
SS26 (wk 28)	-	8.5	8.9	none	NA	-	-	1.0	3.0	ND
SS27 (wk 30)	-	10	8.98	none	NA	871gms Kathon 886	NA	No reading	No reading	Fungus odor present
SS28 (wk 32)	-	8.5	ND	none	NA	-	-	No reading	No reading	ND
SS29 (wk 34)	-	10	ND	none	NA	-	-	No reading	No reading	ND
SS30 (wk 36)	-	11	9.3	none	NA	871 gms Kathon 886	NA	1.0	3.0	Fungus odor
SS31(wk 38)	10 gal of Klear	11	9.3	none	NA	-	-	1.0	3.0	ND

	kool 3089									
SS32 (wk 40)	-	10.5	9.5	none	NA	-	-	1.0	3.0	ND
SS33 (wk 42)	20 gal of Klear kool 3089	10.5	ND	none	NA	-	-	1.0	3.0	ND
SS34 (wk 44)	30 gal of Klear kool 3089	11.0	9.5	none	NA	none	-	1.0	3.0	ND
SS35 (wk 46)	-	12.5	9.2	none	NA	none	-	1.0	3.0	ND
SS36(wk 48)	-	11	9.0	none	NA	none	-	1.0	3.0	ND
SS37(wk 50)	-	12.0	7.97	none	NA	845 gms Kathon 886	NA	3.0	6.0	ND

NA –not available ND – not done
